# Immune checkpoint silencing using RNAi-incorporated nanoparticles enhances antitumor immunity and therapeutic efficacy compared with antibody-based approaches

**DOI:** 10.1136/jitc-2021-003928

**Published:** 2022-02-28

**Authors:** Ji Eun Won, Youngseon Byeon, Tae In Wi, Chan Mi Lee, Ju Hyeong Lee, Tae Heung Kang, Jeong-Won Lee, YoungJoo Lee, Yeong-Min Park, Hee Dong Han

**Affiliations:** 1 Department of Immunology, Konkuk University School of Medicine, Chungju, The Republic of Korea; 2 Department of Obstertrics and Gynecology, Samsung Medical Center, Sungkyunkwan University, Seoul, The Republic of Korea; 3 Department of Integrative Bioscience and Biotechnology, Sejong University, Gwangjin-gu, The Republic of Korea

**Keywords:** vaccination, tumor microenvironment, immunotherapy, adoptive

## Abstract

**Background:**

Cytotoxic CD8^+^ T cell-based cancer immunotherapy has been extensively studied and applied, however, tumor cells are known to evade immune responses through the expression of immune checkpoints, such as programmed death ligand 1 (PD-L1). To overcome these issues, antibody-based immune checkpoint blockades (eg, antiprogrammed cell death 1 (anti-PD-1) and anti-PD-L1) have been revolutionized to improve immune responses. However, their therapeutic efficacy is limited to 15%–20% of the overall objective response rate. Moreover, PD-L1 is secreted from tumor cells, which can interrupt antibody-mediated immune reactions in the tumor microenvironment.

**Methods:**

We developed poly(lactic-co-glycolic acid) nanoparticles (PLGA-NPs) encapsulating PD-L1 small interfering RNA (siRNA) and PD-1 siRNA, as a delivery platform to silence immune checkpoints. This study used the TC-1 and EG7 tumor models to determine the potential therapeutic efficacy of the PLGA (PD-L1 siRNA+PD-1 siRNA)-NPs, on administration twice per week for 4 weeks. Moreover, we observed combination effect of PLGA (PD-L1 siRNA+PD-1 siRNA)-NPs and PLGA (antigen+adjuvant)-NPs using TC-1 and EG7 tumor-bearing mouse models.

**Results:**

PLGA (PD-L1 siRNA+PD-1 siRNA)-NPs boosted the host immune reaction by restoring CD8^+^ T cell function and promoting cytotoxic CD8^+^ T cell responses. We demonstrated that the combination of NP-based therapeutic vaccine and PLGA (siRNA)-NPs resulted in significant inhibition of tumor growth compared with the control and antibody-based treatments (p<0.001). The proposed system significantly inhibited tumor growth compared with the antibody-based approaches.

**Conclusion:**

Our findings suggest a potential combination approach for cancer immunotherapy using PLGA (PD-L1 siRNA+PD-1 siRNA)-NPs and PLGA (antigen+adjuvant)-NPs as novel immune checkpoint silencing agents.

## Background

Cancer immunotherapy is an exciting therapeutic approach that has seen tremendous advances in recent years for various types of cancer.^1^ These approaches have focused on improving the immunological function of cytotoxic T cells.^2^ Among novel immunotherapeutic strategies, immune checkpoint inhibitors such as antibody-based programmed death ligand 1 (anti-PD-L1) and programmed cell death 1 (anti-PD-1) have shown effectiveness against a large number of cancer types.^3^ PD-L1 is expressed on the surface of various cells, including macrophages and dendritic cells (DCs).^4^ In particular, PD-L1 is abundantly expressed in various tumor cells such as lung,^5^ colon,^6^ melanoma,^7^ and leukemic cells,^8^ and contributes to immune escape through its interaction with PD-1 on cytotoxic T cells.^2^ Moreover, recent studies have revealed the intrinsic expression of PD-1 in tumor cells. PD-1 can activate the expression of PD-L1 in tumor cells by means of cross-reactive stimulation, leading to the promotion of cell growth regardless of adaptive immunity.^9 10^

Although anti-PD-L1 or anti-PD-1 blockade is currently approved to treat cancers, the overall response rates are limited to <20% of patients.^11^ More importantly, PD-L1 and PD-1 can be secreted from tumor cells into the tumor microenvironment in a soluble form, which may lead to reduced therapeutic efficacy for antibody blockades.^12^ The immunosuppressive function of secreted PD-L1 in blood circulation has been highly correlated with poor prognosis in multiple cancers.^13^ These secreted PD-L1 increase the complexity and diversity of the PD-1/PD-L1 signaling pathway composition.^12^ Eventually, the secretion of PD-1 or PD-L1 from tumor cells or T cells competitively interrupts the neutralizing activity of antibody-based blockade and induces resistance.^14^ To overcome these hurdles, we hypothesized that immune checkpoint silencing might be a better strategy for enhancing therapeutic efficacy than immune checkpoint blocking.

Therefore, in this study, we propose a small interfering RNA (siRNA)-based immune checkpoint silencing system. The advantages of the siRNA approach include target-specific gene silencing compared with other small molecules or antibody-based approaches.^15^ Despite the therapeutic potential of siRNA, siRNA delivery has led to issues in clinical applications due to its rapid degradation after intravenous injection. Therefore, an effective delivery platform is essential for the use of siRNA.^16^ We selected the poly(lactic-co-glycolic acid) (PLGA) nanoparticle (NP) system as the siRNA delivery platform, which is a particularly attractive option for clinical and biological applications, because of its low toxicity, low immunogenicity, biocompatibility, and biodegradability.^17 18^

To extend our concept, we combined the PLGA-NP-based therapeutic vaccine system with tumor antigens and adjuvants.^19 20^ This approach was selected because of the increased efficiency of intracellular delivery of tumor antigens and adjuvants to DCs, induction of DC maturation, and activation of cytotoxic CD8^+^ T cells via antigen-specific cross-presentation, leading to increased tumor-specific cytotoxic CD8^+^ T cell responses.

In the present study, we suggest novel strategies for siRNA-based immune checkpoint silencing systems that inhibit the expression of secreted PD-L1 in the tumor microenvironment. Moreover, the PLGA-NP-based vaccine system enhanced additional antigen-specific CD8^+^ T cell responses, leading to increased synergistic antitumor responses. These approaches provide a novel combination for cancer immunotherapy that resulted in improved therapeutic efficacy.

## Materials and methods

### Materials

PLGA (resomer RG502H, monomer ratio: 50:50, molecular weight (MW): 7–17 kDa), polyvinyl alcohol (PVA, 80% hydrolyzed, MW: 9–10 kDa), ovalbumin (OVA), polyinosinic-polycytidylic acid sodium salt (poly I:C), and poly-L-lysine (PLL) were purchased from Sigma-Aldrich (St. Louis, Missouri, USA). All other materials were of analytical grade and were used without further purification. The CD8a^+^ T cell isolation kit was purchased from Miltenyl Biotec (Bergisch Gladbach, Germany). Carboxyfluorescein diacetate succinimidyl ester (CFSE) and SYTOX Green were purchased from Thermo Scientific (Waltham, Massachusetts, USA). Antimouse PD-1 (CD279) and antimouse PD-L1 (B7-H1) were purchased from Bio X Cell (West Lebanon, New Hampshire, USA). Recombinant mouse interferon-gamma (IFN-γ) and recombinant human IFN-γ were purchased from ProSpec (Rehovo, Israel). Fluorescein isothiocyanate (FITC)-mIFN-γ and PE-mPD-1 antibody, as well as, IFN-γ and interleukin-2 (IL-2) ELISA kits were purchased from eBioscience (San Diego, California, USA). APC-conjugated CD4, CD8, CD11c, F4/80, mPD-L1, hPD-L1, and PE-CD3 antibodies were purchased from BioLegend (San Diego, California, USA). The PD-L1 ELISA kit was purchased from R&D Systems (Minneapolis, Minnesota, USA). Cy5-conjugated granzyme B and FITC-conjugated CD107a antibodies were purchased from Bioss (Woburn, Massachusetts, USA). CD3 and CD28 antibodies were purchased from BD Biosciences (San Jose, California, USA). PD-1 and PD-L1 antibodies were purchased from Novus (Littleton, Colorado, USA). CD8, Ki67, and matrix metalloproteinase-9 (MMP-9) antibodies were purchased from Abcam (Cambridge, UK). The proliferating cell nuclear antigen (PCNA) antibody was purchased from Cusabio (Houston, Texas, USA). Terminal deoxynucleotidyl transferase dUTP nick end labeling (TUNEL) assay kit was purchased from TREVIGEN (TACS 2 TdT DAB Kit, Gaithersburg, Maryland, USA). RPMI 1640 and fetal bovine serum (FBS) were acquired from Biowest (Nuaille, France). Opti-MEM and hanks balanced salt solution (HBSS) were acquired from Thermo Fisher Scientific (Waltham).

### Preparation of PLGA (PD-L1 siRNA+PD-1 siRNA)-NPs

We prepared PLGA (PD-L1 siRNA+PD-1 siRNA)-NPs using a water-in-oil-in-water (w/o/w) double emulsion solvent evaporation method.^21^ Briefly, 125 μg of PD-1 siRNA, 125 μg of PD-L1 siRNA, and 187.5 μg of PLL were dissolved in 0.2 mL of RNase-free water. This mixture was added dropwise to 2 mL of chloroform containing 40 mg of PLGA using a probe-type sonicator (Sonics & Materials) at 4°C for 1 min (20 pulses of 5 s, with a 3 s gap). The primary emulsion was further emulsified with a secondary water phase (10 mL of 1.0% w/v PVA) at 4°C for 10 min. The chloroform was evaporated using a rotary evaporator at 25°C under vacuum. After evaporation, the PLGA-NPs were washed three times by means of centrifugation at 13,000 rpm for 20 min, and stored at 4°C until use.

PLGA-NP vaccine containing both antigen (OVA or E7 peptide) and adjuvant (poly I:C) was prepared using the w/o/w double emulsion solvent evaporation method. Briefly, 1 mg antigen (OVA or E7 peptide) and 2 mg poly I:C were dissolved in 0.2 mL deionized water and mixed with 2 mL chloroform containing 40 mg PLGA using a probe-type sonicator, at 4°C for 1 min (20 pulses of 5 s, with a 3 s gap). The primary emulsion was further emulsified with a secondary aqueous phase (10 mL of 1.0% w/v PVA) at 4°C for 10 min, to form a secondary emulsion. To completely remove the chloroform, the emulsion was evaporated using a rotary evaporator, at 25°C under vacuum. After evaporation, the suspension of PLGA-NPs was washed three times with deionized water at 4°C by means of centrifugation at 13,000 rpm for 20 min.

The loading efficiency of FITC-labeled siRNA into PLGA-NPs and release behavior were determined using a fluorescence spectrophotometer (RF-5310PC, Shimadzu) at 488 nm.^21^ The size and surface charge of the PLGA-NPs were measured using dynamic light scattering with an electrophoretic light scattering photometer (SZ-100, Horiba).^22 23^ The morphology of PLGA-NPs was observed using scanning electron microscopy (SEM, Inspect F50, FEI, Hillsboro).

### Cell lines and siRNA

TC-1 cells expressing HPV16 and HPV-E7 proteins and OVA-expressing EG7 cells (EL4 cell line transfected with the gene encoding OVA) were cultured in RPMI 1640 medium supplemented with 10% FBS and 0.1% gentamycin. PD-1 siRNA (sense: CACUUCUAGGGACUUGAGA, antisense: UCUCAAGUCCCUAGAAGUG), PD-L1 siRNA (sense: GACUCAAGAUGGAACCUGA, antisense: UCAGGUUCCAUCUUGAGUC), and control siRNA (sense: TTCTCCGAACGTGTCACGT, antisense: ACGTGACACGTTCGGAGAA) were purchased from Sigma-Aldrich.

### Stimulation of CD8^+^ T cells and tumor cells

CD8^+^ T cells were isolated from splenocytes of C57BL/6 mice using a CD8a^+^ T cell isolation kit with AutoMACS purification, and confirmed by means of staining with PE-anti-CD3 and APC-anti-CD8.^24^ The CD8^+^ T cells were stimulated in 24-well plates coated with 0.5 μg/mL anti-CD3 and 5 μg/mL anti-CD28 in phosphate-buffered saline (PBS), overnight at 4°C, and washed twice with PBS prior to use. CD8^+^ T cells were incubated for 24 hours at 37°C in an incubator containing 5% CO.^2^ Finally, we chose CD8^+^ T cells that showed expression of PD-1 by means of PE-anti-PD-1 staining. TC-1 tumor cells were stimulated in 6-well plates with the addition of 10 nM recombinant mouse IFN-γ for 24 hours at 37°C in an incubator containing 5% CO.^2^ Tumor cells were determined by assessing the expression of PD-L1 using APC-anti-PD-L1 staining.

### Intracellular delivery of PLGA (FITC-siRNA)-NPs

Prior to testing the intracellular delivery of PLGA-NPs, we incorporated FITC-siRNA into PLGA-NPs. Briefly, CD8^+^ T cells, DCs, and tumor cells (TC-1 and EG7) were incubated in RPMI 1640 containing 10% FBS with PLGA (FITC-siRNA)-NPs for 30 min at 37°C. After incubation, the intracellular delivery efficiency of PLGA (FITC-siRNA)-NPs was determined using flow cytometry (FACSCalibur with CELLQuest software; BD Biosciences). Additionally, we observed intracellular delivery using confocal microscopy (LSM710, Carl Zeiss). The cells were incubated with PLGA (rhodamine-siRNA)-NPs for 30 min at 37°C, washed with PBS, fixed with 4% (w/v) paraformaldehyde for 10 min at 25°C, and stained with 1 mM SYTOX Green in PBS for 10 min.

### Immune checkpoint silencing using PLGA (PD-L1 siRNA+PD-1 siRNA)-NPs

Prior to determining the silencing effect, CD8^+^ T cells were stimulated using anti-CD3 and anti-CD28 to increase PD-1 expression on CD8^+^ T cell surfaces. TC-1 tumor cells were stimulated with IFN-γ to induce PD-L1 expression. Following that, CD8^+^ T cells (5×10^6^) were incubated with PLGA (PD-L1 siRNA+PD-1 siRNA)-NPs (2 μg of PD-1 siRNA and 2 μg of PD-L1 siRNA) in Opti-MEM medium. TC-1 cells or DCs (5×10^5^) were seeded into 6-well plates and incubated overnight. The cells were transfected with PLGA (PD-L1 siRNA+PD-1 siRNA)-NPs (2 μg of PD-1 siRNA and 2 μg of PD-L1 siRNA) in Opti-MEM. The effect of immune checkpoint silencing was determined using western blot analysis and flow cytometry.

TC-1 and EG7 cells were stimulated with IFN-γ to induce PD-L1 expression. Cells (5×10^5^) were seeded into 6-well plates and incubated overnight. The cells were treated with PLGA (PD-L1 siRNA)-NPs (2 μg of PD-L1 siRNA) in Opti-MEM for 72 hours. The tumor-conditioned media (TCM) was collected and centrifuged at 1500 rpm for 3 min. Secreted PD-L1 expression in tumor cells was determined by measuring its levels in the TCM using western blot analysis.

Female C57BL/6 mice (5–6 weeks old, 20 g) were purchased from Orient Bio (Gapyeong, South Korea).

To generate tumors, TC-1 cells (1×10^6^ cells per 0.1 mL HBSS) were injected subcutaneously into C57BL/6 mice. To confirm the presence of circulating PD-L1 in TC-1 tumor-bearing mice, PLGA (PD-L1 siRNA)-NPs (5 μg of PD-L1 siRNA) were intravenously injected into the mice twice in a week. On day 7, serum and tumor tissues were collected and analyzed for PD-L1 using ELISA.

### Migration assay of tumor cells

Prior to the TC-1 cell migration assay, TC-1 cells (3×10^5^) were seeded into 6-well plates and incubated overnight. After making a scratch, the TC-1 cells were further incubated with PLGA (PD-L1 siRNA+PD-1 siRNA)-NPs (2 μg of siRNA) or anti-PD-L1+anti-PD-1 (2 μg of antibody). After 24 hours, the tumor cell migration was confirmed, and the level of PD-1, PD-L1, MMP-9, and PCNA in the tumor cells were confirmed using western blot analysis.

### Proliferation assay for CD8^+^ T cells

Prior to the CD8^+^ T cell proliferation assay, CD8^+^ T cells and TC-1 cells were stimulated to induce PD-1 and PD-L1 expression, following which PD-1 and PD-L1 were silenced using PLGA (PD-L1 siRNA+PD-1 siRNA)-NPs. CD8^+^ T cells were labeled with CFSE, and then seeded into 48-well plates at a cell density of 1×10^6^ cells/well. TC-1 cells were added to the CFSE-labeled CD8^+^ T cells at various E:T ratios (where effector: CD8^+^ T cell and target: TC-1 cells; 1:1, 1:5, 1:10, 1:50, and 1:100). After 48 hours of incubation, the proliferation of CD8^+^ T cells was examined using flow cytometry.^25^

### Activation of CD8^+^ T cells

To determine the functional effect of PD-L1 secreted from TC-1 against CD8^+^ T cells, TC-1 cells were stimulated with IFN-γ to express PD-L1. Next, 2 μg of anti-PD-L1 or 2 μg of PLGA (PD-L1 siRNA)-NPs was added to the cells and they were incubated for 3 days. At the end of the incubation, the TCM was collected and centrifuged at 1500 rpm for 3 min. The supernatant was collected and stored at −70°C. CD8^+^ T cells (1×10^6^ cells/well) stimulated using anti-CD3 and anti-CD28 were seeded into 48-well plates. RPMI 1640 medium alone was used as a negative control, to which TCM was added. After 24 hours incubation, IFN-γ and IL-2 secreted from CD8^+^ T cells were measured using ELISA, and the expression levels of AKT and p-AKT in CD8^+^ T cells were measured using western blot analysis. In addition, the activation of CD8^+^ T cells was determined in terms of the expression levels of granzyme B and CD107a in CD8^+^ T cells, which were assessed using flow cytometry.

### Antitumor efficacy of PLGA (PD-L1 siRNA+PD-1 siRNA)-NPs

Prior to measuring the therapeutic efficacy, we assessed the biodistribution of PLGA (Cy5)-NPs after injection into TC-1 tumor-bearing mice. To generate tumors, TC-1 cells (1×10^6^ cells/0.1 mL HBSS) were subcutaneously injected into C57BL/6 mice. After growing the tumor tissue, PLGA (Cy5)-NPs were intravenously injected into the mice (n=3/group). Cy5 is a fluorescent, which was used as a model drug. After 24 hours, biodistribution of PLGA (Cy5)-NP into tumor-bearing mice was monitored using in vivo imaging system (Perkin Elmer, Waltham, Massachusetts, USA) at the appropriate wavelength (λex=680 nm and λem=740 nm). The emitted signals were collected using a time-correlated single-photon counting system software (Living image 4.5.5).

We next evaluated the therapeutic efficacy of PLGA-NPs in TC-1 and EG7 tumor-bearing mice. To generate tumors, TC-1 cells (1×10^6^ cells/0.1 mL HBSS) or EG7 cells (1×10^6^ cells/0.1 mL HBSS) were subcutaneously injected into C57BL/6 mice. The mice (n=5/group) were monitored daily for adverse effects of treatment and euthanized when the control group seemed moribund. The PLGA (E7+poly I:C)-NP or PLGA (OVA+poly I:C)-NP vaccine used in the previous study was injected to induce a tumor antigen-specific immune response.^22^ The PLGA (E7+poly I:C)-NP (E7 and poly I:C; 0.3 mg/kg of each) or PLGA (OVA+poly I:C)-NP (OVA and poly I:C; 5 mg/kg of each) vaccine was administered once per week for 2 weeks by means of subcutaneous injection. Anti-PD-1, anti-PD-L1, anti-PD-1+anti-PD-L1, PLGA (PD-L1 siRNA)-NPs, PLGA (PD-1 siRNA)-NPs, and PLGA (PD-L1 siRNA+PD-1 siRNA)-NPs were then administered twice per week for 4 weeks by means of intravenous injection at a dose of 200 μg/kg of each antibody or siRNA. Tumor volume and survival of the mice were recorded. The tumor volume was measured using calipers, and the volume was calculated using the following formula^26^:

$$Tumorvolume(mm^{3})=length\times (width{)}^{2}/2$$

Tumor volume, tumor weight in the mice, and images of tumor-bearing mice were recorded. The investigators who performed the necropsies, tumor collection, and tissue processing were blinded to the treatment groups. Tissue specimens were fixed with either 4% paraformaldehyde or optimum cutting temperature compound.

### Immunohistochemical staining

Procedures for immunohistochemical analysis of PD-L1 (anti-PD-L1) and PD-1 (anti-PD-1) expression, CD8^+^ T cell population (anti-CD8), and cell proliferation (anti-Ki67) were performed.^22 27^ All of these analyses were recorded in five random fields per slide. The bar graph indicates the percentage of positive (brown)/total tumor cells (blue) in the same tissue area. In addition, TUNEL assay was performed to determine cell apoptosis.^22^ Apoptotic cells were quantified by counting the number of apoptotic cells in five random fields of each slide at ×400 magnification.

### Biochemical toxicity of PLGA (PD-L1 siRNA+PD-1 siRNA)-NPs

Female C57BL/6 mice (5–6 weeks old, 20 g) were grouped as follows (n=3/group): (1) control and (2) PLGA (PD-L1 siRNA+PD-1 siRNA)-NPs. Mice in the PLGA (PD-L1 siRNA+PD-1 siRNA)-NPs group were intravenously injected with a single treatment of the same therapeutic dose. To determine the biochemical toxicity of the PLGA (PD-L1 siRNA+PD-1 siRNA)-NPs, the levels of aminotransferase (AST), alanine aminotransferase (ALT), and blood urea nitrogen (BUN) were analyzed using diagnostic kits (Roche, Basel, Switzerland). Blood samples were collected from the mice through the retro-orbital sinus on days 1 and 7 after injection of PLGA (PD-L1 siRNA+PD-1 siRNA)-NPs. Serum was obtained from the blood by means of centrifugation at 3000 rpm, 4°C for 10 min.

### Statistical analysis

Differences in continuous variables were analyzed using Student’s t-test, for comparison between two groups, while analysis of variance was performed to assess differences among multiple groups. The Mann-Whitney rank-sum test was performed for values that were not normally distributed. The Statistical Package for the Social Sciences version 22 was used for all calculations. Differences were considered statistically significant at p<0.05.

## Results

### Characteristics of PLGA (PD-L1 siRNA+PD-1 siRNA)-NPs

**Figure 1:**
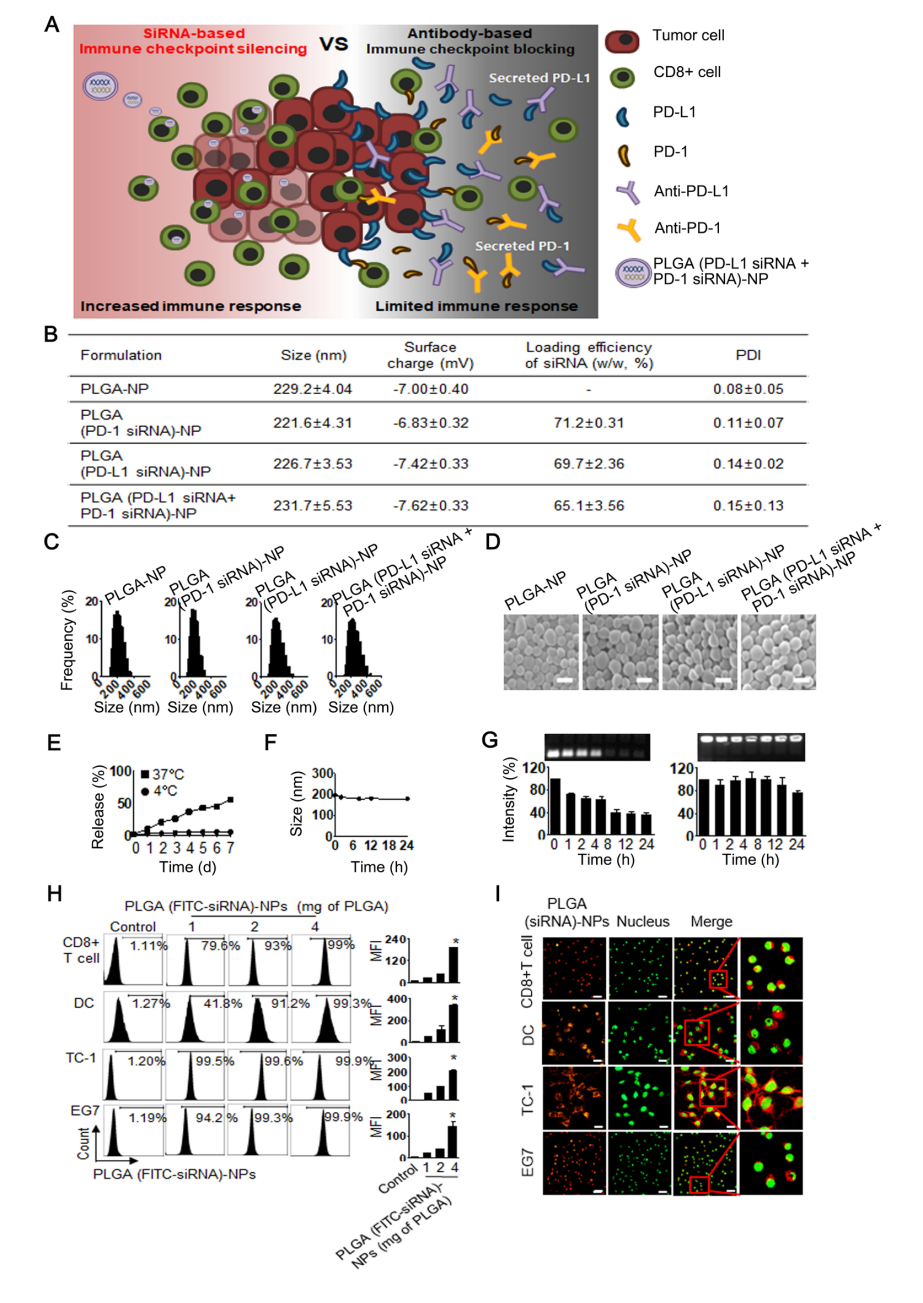
Physical properties of poly(lactic-co-glycolic acid) (PLGA) (small interfering RNA (siRNA))-nanoparticles (NPs). (A) A conceptual scheme of this study, which aimed at immune checkpoint silencing using a siRNA-based nanoparticle platform. (B) Physical characteristics of PLGA (siRNA)-NPs. (C) Size distribution and (D) morphology of PLGA (siRNA)-NPs (scale bar: 200 nm). (E) Release of siRNA from PLGA (siRNA)-NPs in phosphate-buffered saline at 4°C or 37°C. (F) Stability of PLGA (siRNA)-NPs in 50% serum at 37°C. (G) Electrophoresis of PLGA (siRNA)-NPs in 50% serum. Intracellular delivery efficiency of PLGA (siRNA)-NPs in CD8^+^ T cells, dendritic cells (DCs), TC-1, and EG7 cells. The cells were incubated for 30 min at 37°C. (H) Flow cytometry analysis for PLGA (fluorescein isothiocyanate (FITC)-siRNA)-NPs. (I) Confocal microscopic images for PLGA (rhodamine-siRNA)-NPs. Red color indicates rhodamine-siRNA, while green indicates the nuclei of the cells (scale bar: 20 μm). Error bars represent SEM. *P<0.001.

In this study, we prepared PLGA-NPs incorporating an siRNA system for target gene silencing of immunosuppressive PD-L1 and PD-1. This siRNA-based immune checkpoint silencing system could fundamentally inhibit the secretion of PD-L1 from tumor cells into the tumor microenvironment, leading to an increased immune response compared with antibody-based blockades (figure 1A). We first determined the physical properties of the PLGA (PD-L1 siRNA+PD-1 siRNA)-NPs. Mean particle size and surface charge of PLGA-NPs were around 231±5.53 nm and –7.62±0.33 mV, respectively. The loading efficiency of both PD-1 siRNA and PD-L1 siRNA into PLGA-NPs was up to 65.1%, while the polydispersity index was 0.15±0.13 (figure 1B). Representative histograms of the size distribution and spherical morphologies, as observed using SEM and a particle size analyzer, are given in figure 1C, D. The release of siRNA from PLGA (siRNA)-NPs showed a weak increase at 4°C, however, siRNA showed sustained release of up to 56% for 7 days at 37°C (figure 1E). Moreover, the size of PLGA (siRNA)-NP was maintained in 50% serum solution at 37°C, indicating that PLGA (siRNA)-NP was stable in serum (figure 1F). Next, we determined the stability of siRNA in a 50% serum solution using electrophoresis. Although naked siRNA degraded with incubation time, encapsulation of the siRNA with PLGA (siRNA)-NP protected against degradation (figure 1G). In addition, the physical properties of PLGA (OVA+poly I:C)-NPs and PLGA (E7+poly I:C)-NPs used in this study as therapeutic NP-based vaccines are shown in online supplemental figure S1.

10.1136/jitc-2021-003928.supp1 Supplementary data

### Intracellular delivery of PLGA (siRNA)-NPs

We next assessed the intracellular delivery of PLGA-NPs incorporating FITC-labeled siRNA using flow cytometry and confocal microscopy (figure 1H, I). The intracellular delivery efficiency of PLGA (FITC-siRNA)-NPs increased in a dose-dependent manner in immune cells (CD8^+^ T cells and DCs) and tumor cells (TC-1 and EG7) (p<0.001, figure 1H). We observed the intracellular delivery of PLGA (rhodamine-siRNA)-NPs in various cells to confirm siRNA delivery using confocal microscopy, resulting that siRNA showed effective intracellular delivery in both immune cells and tumor cells (figure 1I). In addition, intracellular delivery of PLGA (Cy5)-NPs without siRNA incorporation was confirmed (online supplemental figure S2).

**Figure 2:**
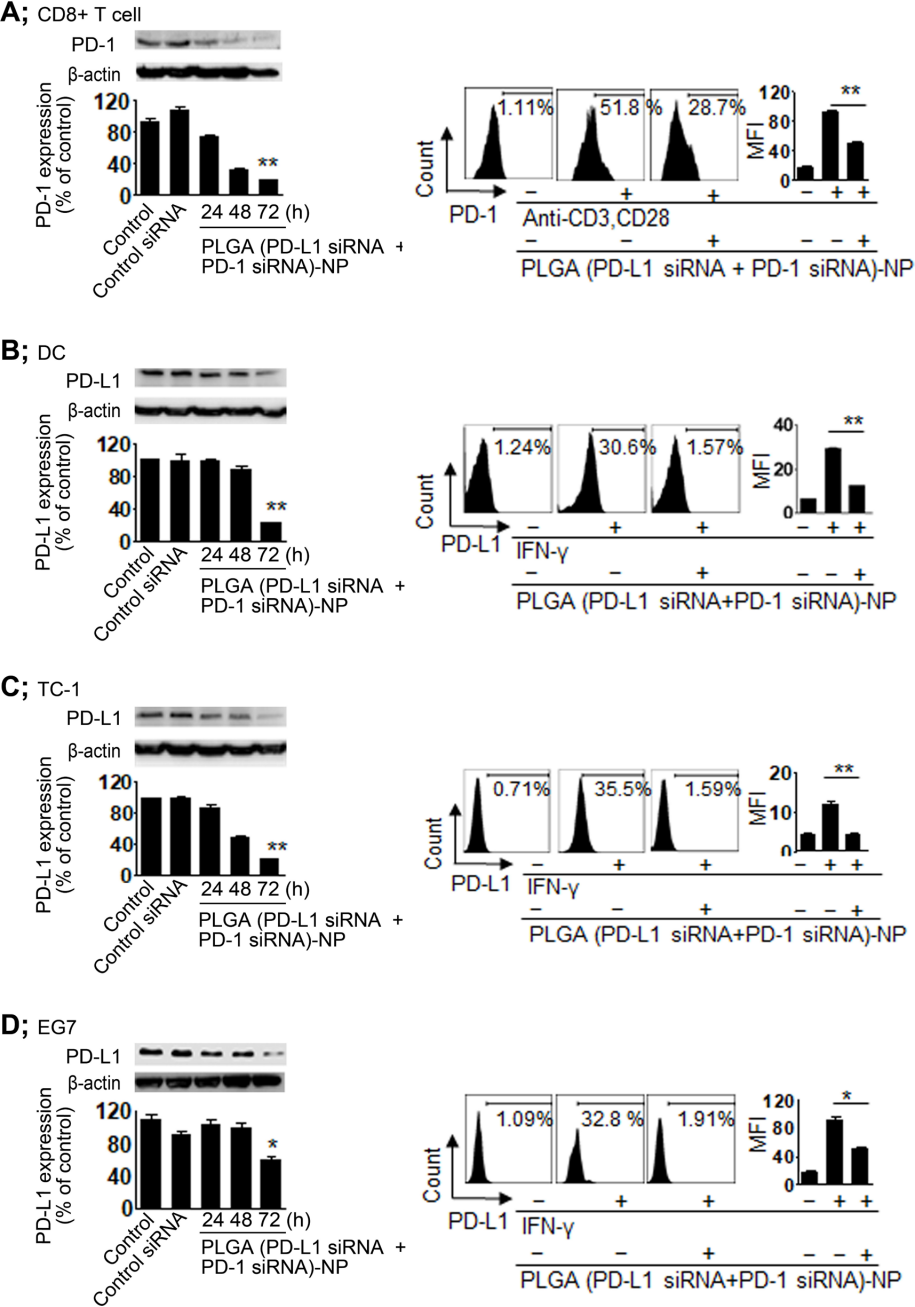
Programmed cell death 1 (PD-1) and programmed death ligand 1 (PD-L1) silencing using poly(lactic-co-glycolic acid) (PLGA) (small interfering RNA (siRNA))-nanoparticles (NPs). (A) PD-1 silencing in CD8^+^ T cells was assessed using western blot analysis and flow cytometry. CD8^+^ T cells were stimulated with anti-CD3 and anti-CD28 for 24 hours to increase PD-1 expression and then treated with PLGA (PD-L1 siRNA+PD-1 siRNA)-NPs. PD-L1 silencing in (B) dentritic cells (DCs), (C) TC-1 cells, and (D) EG7 cells was assessed using western blot analysis and flow cytometry. The cells were stimulated with interferon (IFN)-γ for 24 hours to increase PD-L1 expression and then treated with PLGA (PD-L1 siRNA+PD-1 siRNA)-NPs. Error bars represent the SEM. *P<0.01 and **p<0.001.

### Immune checkpoint silencing

We performed western blot analysis and flow cytometry analysis to determine the gene silencing effect of PD-1 and PD-L1 in various cells. Prior to confirming immune checkpoint silencing, we induced CD8^+^ T cell activation using both anti-CD3 and anti-CD28 to increase PD-1 expression on the cell surface (online supplemental figure S3A). In addition, we induced PD-L1 expression on the surface of tumor cells by means of IFN-γ stimulation (online supplemental figure S3B). PLGA (PD-L1 siRNA+PD-1 siRNA)-NPs significantly inhibited PD-1 expression in CD8^+^ T cells (p<0.001, figure 2A) as well as PD-L1 expression in DCs (p<0.001), TC-1 (p<0.001), and EG7 (p<0.01) cells compared with those in the control (figure 2B–D).

**Figure 3:**
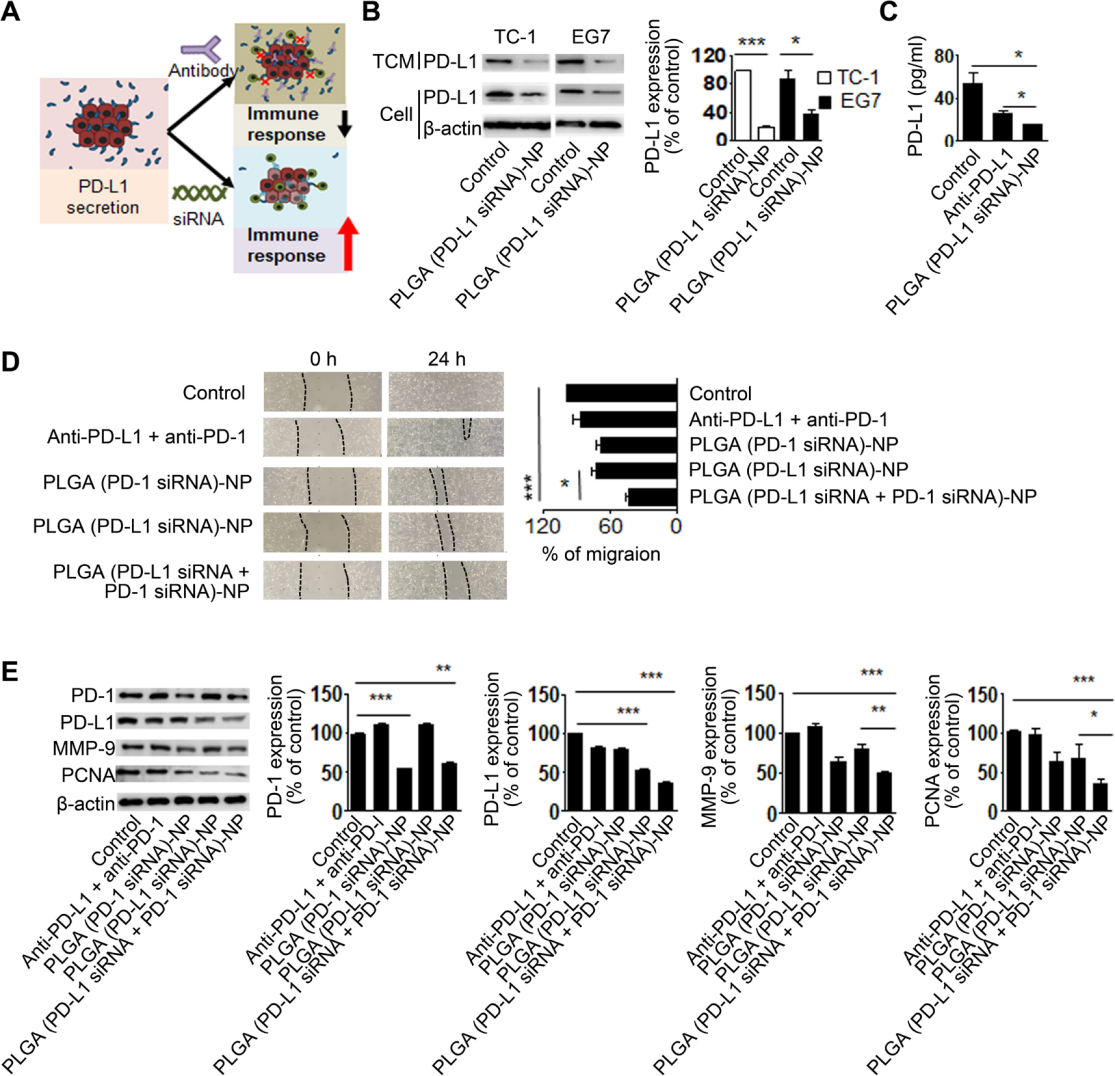
Migration and cell growth after immune checkpoint silencing. (A) An illustration of the strategy for small interfering RNA (siRNA)-based immune checkpoint silencing. (B) Secreted programmed death ligand 1 (PD-L1) in the tumor-conditioned media (TCM) was determined using western blot analysis. Tumor cells were stimulated with interferon (IFN)-γ for 24 hours to increase PD-L1 expression on their surface membrane. The tumor cells were incubated with poly(lactic-co-glycolic acid) (PLGA) (PD-L1 siRNA)-NPs for 72 hours. (C) Seven days after injection of PLGA (PD-L1 siRNA)-nanoparticles (NPs) into TC-1 tumor-bearing mice, the mice were sacrificed, and blood was collected from them to obtain serum. PD-L1 levels in the serum were evaluated using ELISA. (D) TC-1 cell growth and migration after PLGA (PD-L1 siRNA+programmed cell death 1 (PD-1) siRNA)-NPs treatment for 24 hours. (E) Cell growth and migration analysis were assessed using western blot analysis. Error bars represent the SEM. *P<0.05, **p<0.01, and ***p<0.001.

### Migration and cell growth after immune checkpoint silencing of tumor cells

In this study, we hypothesized that antibody-based approaches might be less effective than siRNA approaches because antibody blockades can bind to the secreted PD-L1, which leads to the suppression of therapeutic outcomes (figure 3A). Based on our study conceptualization, we observed the secretion of PD-L1 from tumor cells and anticipated that the use of siRNA might prevent PD-L1 secretion. PD-L1 expression was found in the TCM from untreated cells, whereas weak PD-L1 expression was found in both TC-1 cells and the TCM after treatment with PLGA (PD-L1 siRNA)-NPs (p<0.001, figure 3B). This result clearly indicates that PD-L1 silencing by PD-L1 siRNA can prevent the secretion of PD-L1 from tumor cells. Moreover, to determine the secretion of PD-L1 into the bloodstream, we analyzed PD-L1 circulation after PLGA (PD-L1 siRNA)-NP treatment. When PLGA (PD-L1 siRNA)-NPs were injected into TC-1 tumor-bearing mice by means of intravenous injection, there was a significant decrease in the PD-L1 circulation in the bloodstream, as compared with that in the control and anti-PD-L1-treated groups (p<0.05, figure 3C).

Remarkably, recent studies have found intrinsic PD-1 expression in tumor cells, and PD-1 additionally induces PD-L1 expression in tumor cells through cross-reactive stimulation in the tumor microenvironment, which promotes tumor cell growth and migration.^28^ Therefore, we confirmed cell growth and migration after silencing of both PD-1 and PD-L1 in TC-1 tumor cells. Although anti-PD-1+anti-PD-L1 treatment showed weak inhibition, treatment with PLGA (PD-L1 siRNA+PD-1 siRNA)-NPs showed 53% inhibition of cell growth and migration (p<0.001, figure 3D). Additionally, PLGA (PD-L1 siRNA+PD-1 siRNA)-NP treatment led to a significant decrease in MMP-9 and PCNA expression, as compared with that on control (p<0.001, figure 3E). These results indicated that dual silencing of PD-1 and PD-L1 in tumor cells is more effective in inhibiting cell growth and migration.

### Proliferation and activation of CD8^+^ T cells

**Figure 4:**
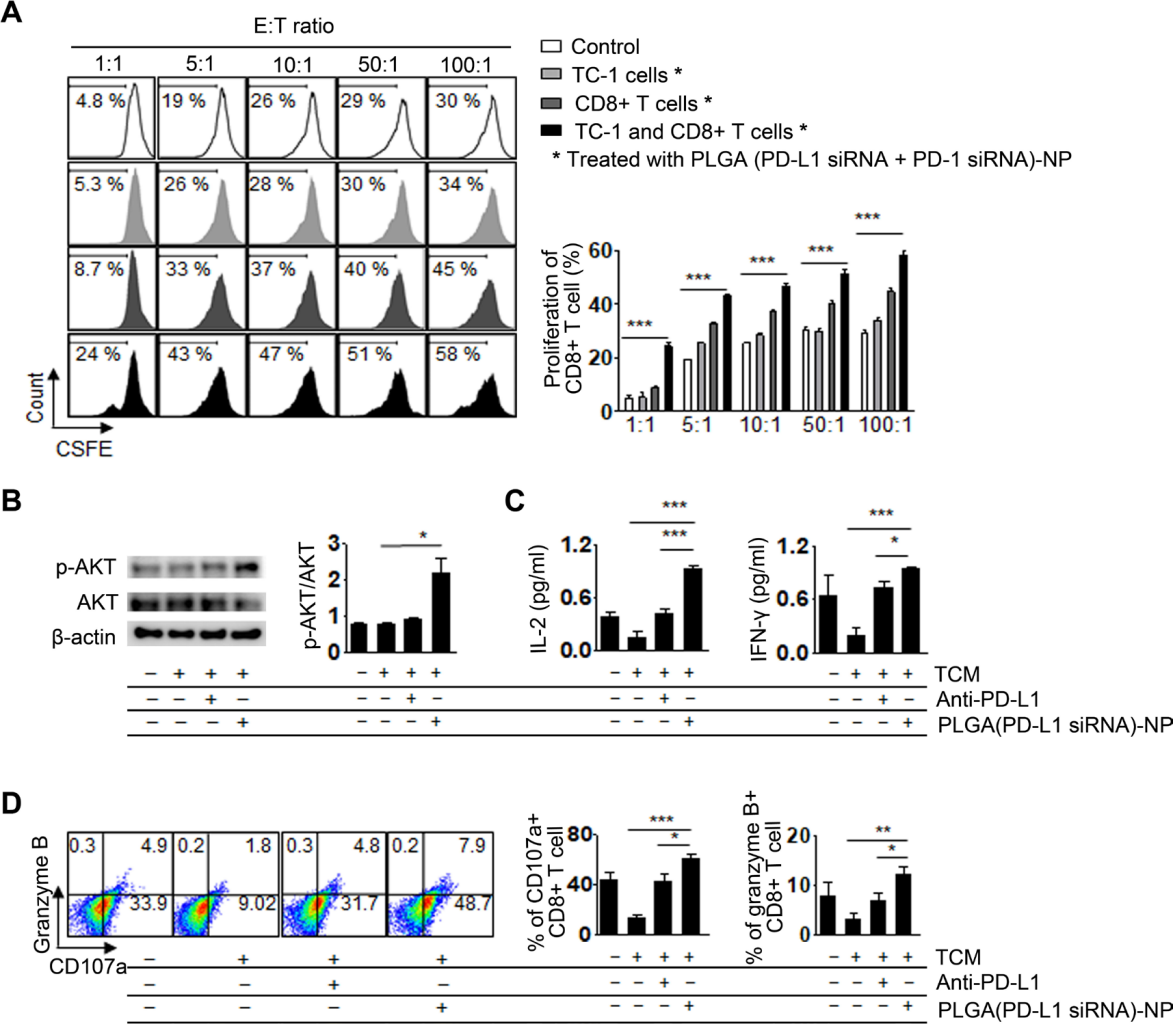
Proliferation and activation of CD8^+^ T cells. (A) Quantitative analysis of proliferating CD8^+^ T cells was carried out using flow cytometry. CD8^+^ T cells were isolated from splenocytes and labeled with carboxyfluorescein diacetate succinimidyl ester (CFSE). The CD8^+^ T cells were stimulated with anti-CD3 and anti-CD28, and then co-cultured with TC-1 cells for 48 hours (effecter: CD8^+^ T cell, target: TC-1). (B) Activation of CD8^+^ T cells in the tumor-conditioned media (TCM) was assessed using western blot analysis. TCM was isolated from TC-1-cultured media after programmed death ligand 1 (PD-L1)-silencing. (C) Pro-inflammatory cytokines in the TCM from CD8^+^ T cells were measured using ELISA. (D) Granzyme B and CD107a in the TCM from CD8^+^ T cells were measured using flow cytometry. Error bars represent the SEM. *P<0.05, **p<0.01, and ***p<0.001.

We next confirmed whether the proliferation of CD8^+^ T cells increased after PD-1 and PD-L1 silencing. After stimulating CD8^+^ T cells using anti-CD3 and anti-CD28, we silenced PD-1 using PLGA (PD-L1 siRNA+PD-1 siRNA)-NPs. Additionally, TC-1 cells were stimulated with IFN-γ to increase PD-L1 expression, following which the PD-L1 in TC-1 cells was silenced using PLGA (PD-L1 siRNA+PD-1 siRNA)-NPs. The PLGA (PD-L1 siRNA+PD-1 siRNA)-NP treatment group showed significantly increased proliferation of CD8^+^ T cells with an increasing E:T ratio (E: effector CD8^+^ T cells, T: target TC-1 cells) compared with that in the control group (p<0.001, figure 4A).

PD-L1 secreted from tumor cells into the tumor microenvironment has been found to decrease CD8^+^ T cell activation.^29^ Therefore, we assessed whether secreted PD-L1 decreased CD8^+^ T cell activation by measuring p-AKT expression because p-AKT is highly associated with CD8^+^ T cell activation and proliferation.^30^ The TCM was prepared by isolating TC-1 culture media after PD-L1 silencing using PLGA (PD-L1 siRNA)-NPs. Subsequently, CD8^+^ T cells were cultured in TCM for 24 hours. There was a significant increase in p-AKT expression in CD8^+^ T cells cultured in PD-L1-silenced TCM as compared with that in control and anti-PD-L1-treated groups (p<0.05), indicating that activation of CD8^+^ T cells may be induced by the increasing p-AKT expression caused by inhibition of PD-L1 secretion from TC-1 tumor cells (figure 4B). Moreover, under the same conditions, we measured levels of the inflammatory cytokines IL-2, IFN-γ, granzyme B, and degranulation marker CD107a as these serve as CD8^+^ T cell activation biomarkers. There was a significant increase (p<0.001) in the levels of IFN-γ, IL-2, granzyme B, and CD107a in CD8^+^ T cells (figure 4C–D), indicating that inhibition of PD-L1 secretion from tumor cells contributed to CD8^+^ T cell activation and could help restore CD8^+^ T cell proliferation.

### Therapeutic efficacy of PLGA (PD-L1 siRNA+PD-1 siRNA)-NPs

**Figure 5:**
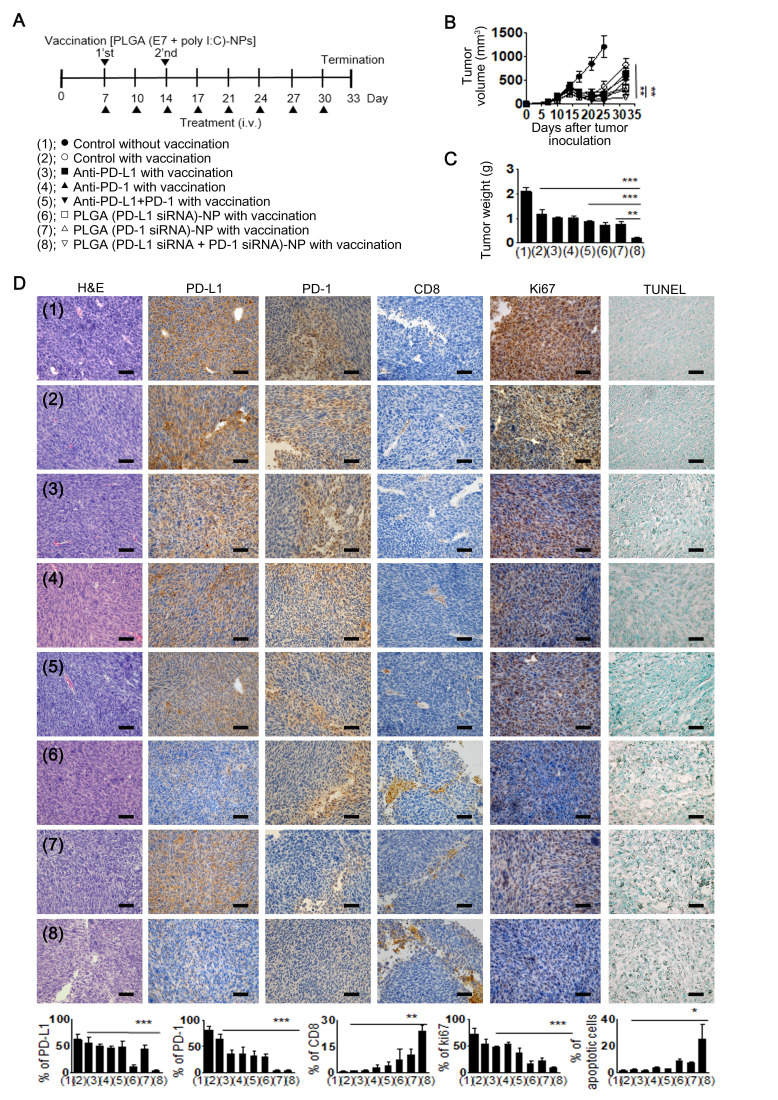
Therapeutic efficacy of poly(lactic-co-glycolic acid) (PLGA) (small interfering RNA (siRNA))-nanoparticles (NPs) against TC-1 tumor-bearing C57BL/6 mouse model. Treatment began 1 week after subcutaneous injection of TC-1 cells into the mice. PLGA (E7+poly I:C)-NPs were subcutaneously injected as a therapeutic vaccine, once per week for 2 weeks. The PLGA (programmed death ligand 1 (PD-L1) siRNA+programmed cell death 1 (PD-1) siRNA)-NPs with vaccination (200 μg/kg of siRNA or antibody) was intravenously injected twice per week for 4 weeks. (A) Experimental schedule for tumor therapy. (B) Tumor volume and (C) tumor weight. (D) Immunohistochemical analysis of TC-1 tumor tissues for PD-L1 (anti-PD-L1) and PD-1 (anti-PD-1) expression, infiltration of CD8^+^ T cells (anti-CD8), cell proliferation (anti-Ki67), and apoptosis (Terminal deoxynucleotidyl transferase dUTP nick end labeling (TUNEL) assay) (scale bar: 50 µm). Error bars represent the SEM. *P<0.05, **p<0.01, and ***p<0.001.

Prior to assessing therapeutic efficacy, we observed the delivery efficiency of PLGA (Cy5)-NPs to the tumor microenvironment on intravenous injection into tumor-bearing mice. The PLGA (Cy5)-NPs showed effective accumulation in the tumor microenvironment (online supplemental figure S4). Based on the results of delivery efficiency, we determined the potential therapeutic efficacy of PLGA (PD-L1 siRNA+PD-1 siRNA)-NPs using the TC-1 tumor model. TC-1 cells were derived from primary lung epithelial cells of C57BL/6 mice and expressed HPV16-E6 and HPV16-E7.^22^ In addition, to extend our concept, we used E7 peptide-incorporated PLGA (E7+poly I:C)-NPs as a therapeutic vaccine to induce an E7-specific CD8^+^ T cell immune response.^31 32^ Vaccination with PLGA (E7+poly I:C)-NPs led to an increased antigen-specific immune response, as has been reported previously.^22^ Seven days after subcutaneous injection of TC-1 tumor cells into C57BL/6 mice, PLGA (E7+poly I:C)-NPs were subcutaneously injected into the mice twice at an interval of 1 week. The mice were then randomly allocated to the following groups (n=5 mice/group): (1) negative control (without vaccination), (2) positive control (with vaccination), (3) anti-PD-L1 with vaccination, (4) anti-PD-1 with vaccination, (5) anti-PD-L1+anti-PD-1 with vaccination, (6) PLGA (PD-L1 siRNA)-NPs with vaccination, (7) PLGA (PD-1 siRNA)-NPs with vaccination and (8) PLGA (PD-L1 siRNA+PD-1 siRNA)-NPs with vaccination. Antibodies (200 μg/kg) and siRNA (200 μg/kg) were intravenously injected into the TC-1 tumor-bearing mice twice in a week for 4 weeks (figure 5A). The PLGA (PD-L1 siRNA+PD-1 siRNA)-NPs with vaccination group showed significant inhibition of tumor growth as compared with the positive control with vaccination (84% decrease, p<0.01) and anti-PD-L1+anti-PD-1 with vaccination groups (74% decrease, p<0.01, figure 5B). Notably, there was a significant reduced in tumor weight in the PLGA (PD-L1 siRNA+PD-1 siRNA)-NPs with vaccination group as compared with that in the positive control with vaccination (83% decrease, p<0.001) and anti-PD-L1+anti-PD-1 with vaccination groups (78% decrease, p<0.001) (figure 5C).

To determine the potential therapeutic mechanisms underlying the efficacy of PLGA (PD-L1 siRNA+PD-1 siRNA)-NPs in tumor tissues, we examined the tumors for PD-L1 (anti-PD-L1) and PD-1 (anti-PD-1) expression, infiltration of CD8^+^ T cells (anti-CD8), cell proliferation (anti-Ki67), and apoptosis (TUNEL assay) (figure 5D). PLGA (PD-L1 siRNA+PD-1 siRNA)-NP treatment significantly inhibited PD-L1 and PD-1 expression (p<0.001), increased CD8^+^ T cell infiltration (p<0.01), reduced cell proliferation (p<0.001), and increased apoptosis (p<0.05) as compared with those in the other groups.

**Figure 6:**
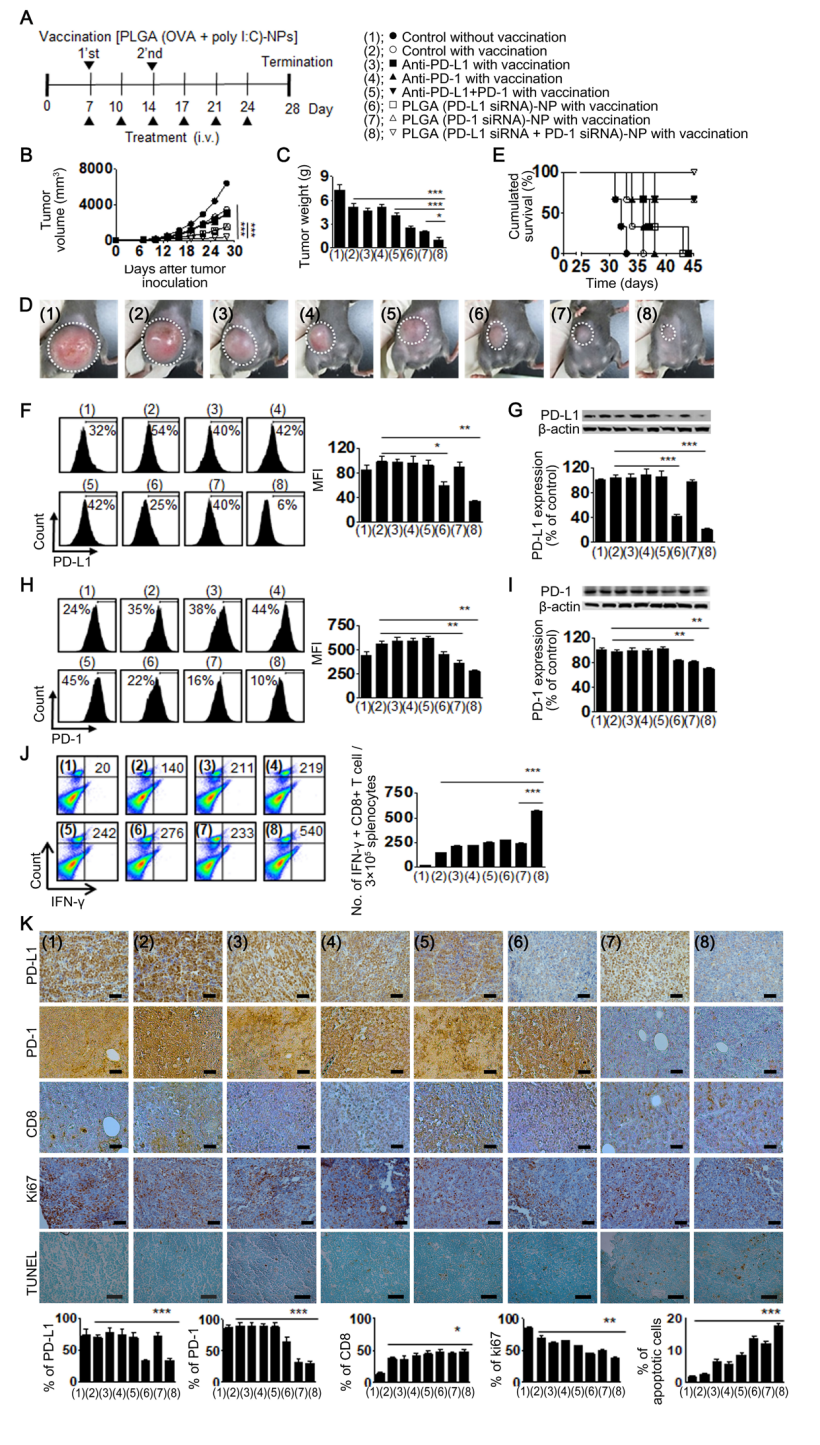
Therapeutic efficacy of poly(lactic-co-glycolic acid) (PLGA) (small interfering RNA (siRNA))-nanoparticles (NPs) against EG7 tumor-bearing mouse model. Treatment began 1 week after subcutaneous injection of EG7 cells into the mice (n=5/group). PLGA (ovalbumin (OVA)+poly I:C)-NPs were administered once per week for 2 weeks by means of subcutaneous injection. The PLGA (programmed death ligand 1 (PD-L1) siRNA+programmed cell death 1 (PD-1) siRNA)-NPs with vaccination group was injected twice per week for 3 weeks by means of intravenous injection (200 μg/kg of siRNA or antibody). (A) Experimental schedule. (B) Tumor volume, (C) tumor weight, and (D) images of mice. (E) Survival of treated mice. (F, G) PD-L1 expression in the tumor tissues was examined using flow cytometry and western blot analysis. (H, I) PD-1 expression in the tumor tissues was examined using flow cytometry and western blot analysis. (J) Cytotoxic CD8^+^ T cells in the splenocytes were counted using flow cytometry. The bar graph indicates the number of CD8^+^ and interferon (IFN)-γ+cells/3×10^5^ splenocytes. (K) Immunohistochemical analysis for expression of PD-L1 (anti-PD-L1) and PD-1 (anti-PD-1), infiltration of CD8^+^ T cell (anti-CD8), cell proliferation (anti-Ki67), and apoptosis (Terminal deoxynucleotidyl transferase dUTP nick end labeling (TUNEL) assay) (scale bar: 50 µm). Error bars represent the SEM. *P<0.05, **p<0.01, and ***p<0.001.

To establish that the therapeutic effects of PLGA (PD-L1 siRNA+PD-1 siRNA)-NPs are not unique to just one target tumor, we used an additional system, OVA-expressing EG7-OVA (EL4 cell line transfected with the gene encoding for OVA) lymphoma cells.^22^ Seven days after subcutaneous injection of EG7 cells into C57BL/6 mice twice in a week, vaccination with PLGA (OVA+poly I:C)-NPs was performed via the subcutaneous route. The mice were then randomly allocated to the following groups (n=5 mice/group): (1) negative control (without vaccination), (2) positive control (with vaccination), (3) anti-PD-L1 with vaccination, (4) anti-PD-1 with vaccination, (5) anti-PD-L1+anti-PD-1 with vaccination, (6) PLGA (PD-L1 siRNA)-NPs with vaccination, (7) PLGA (PD-1 siRNA)-NPs with vaccination, and (8) PLGA (PD-L1 siRNA+PD-1 siRNA)-NPs with vaccination. Therapeutic doses of antibody (200 μg/kg) and siRNA (200 μg/kg) were intravenously injected into EG7 tumor-bearing mice twice in a week for 3 weeks as shown in figure 6A. The PLGA (PD-L1 siRNA+PD-1 siRNA)-NPs with vaccination group showed significant inhibition of tumor growth compared with the positive control with vaccination (89% decrease, p<0.001) and anti-PD-L1+anti-PD-1 with vaccination (87% decrease, p<0.001) groups (figure 6B). Notably, the tumor weight in the PLGA (PD-L1 siRNA+PD-1 siRNA)-NPs with vaccination group was significantly lower than that in the positive control with vaccination (80% decrease, p<0.001) and anti-PD-L1+anti-PD-1 with vaccination (75% decrease, p<0.001) groups (figure 6C, D). Additionally, mice treated with PLGA (PD-L1 siRNA+PD-1 siRNA)-NPs showed a 60% higher survival for at least 50 days as compared with the control and other groups, where all mice died within 45 days (figure 6E).

We next performed flow cytometry and western blot analysis to confirm the expression of PD-L1 and PD-1 in tumor tissues. PD-L1 and PD-1 expression levels were significantly reduced in the PLGA (PD-L1 siRNA)-NPs with vaccination and PLGA (PD-L1 siRNA+PD-1 siRNA)-NPs with vaccination groups as compared with those in the control group (figure 6F–I). Cytotoxic CD8^+^ T cells in splenocytes were significantly increased in the PLGA (PD-L1 siRNA+PD-1 siRNA)-NPs with vaccination group as compared with those in the control group (p<0.001, figure 6J). Additionally, we stained the tumor tissues to examine PD-L1 and PD-1 expression, infiltration of CD8^+^ T cells, cell proliferation, and apoptosis (figure 6K). PLGA (PD-L1 siRNA+PD-1 siRNA)-NPs with vaccination group showed significant inhibition of both PD-L1 (p<0.001) and PD-1 (p<0.001) expression, increased CD8^+^ T cell infiltration (p<0.05), reduced cell proliferation (p<0.01), and increased apoptosis (p<0.001) as compared with those in the control group.

Moreover, we assessed the therapeutic efficacy of the treatment of PLGA (PD-L1 siRNA+PD1 siRNA)-NPs and anti-PD-L1+anti-PD-1 to compare the dose-dependent anticancer effect at high doses of antibody as suggested in a clinical protocol.^33^ The dose of siRNA in PLGA (PD-L1 siRNA+PD-1 siRNA)-NPs was used at 200 μg/kg, which was the same as that used in this study. Anti-PD-L1+anti-PD-1 were used at 10,000 μg/kg, which is accepted as the maximum tolerance dose.^34 35^ According to the protocol, antibodies (anti-PD-L1+anti-PD-1) were injected once every 2 weeks. Although antibody treatment showed therapeutic efficacy at high doses, PLGA (PD-L1 siRNA+PD-1 siRNA)-NPs significantly inhibited tumor growth compared with the antibody treatment, even at low doses (online supplemental figure S5).

### Biochemical toxicity of PLGA (PD-L1 siRNA+PD-1 siRNA)-NPs

**Figure 7:**
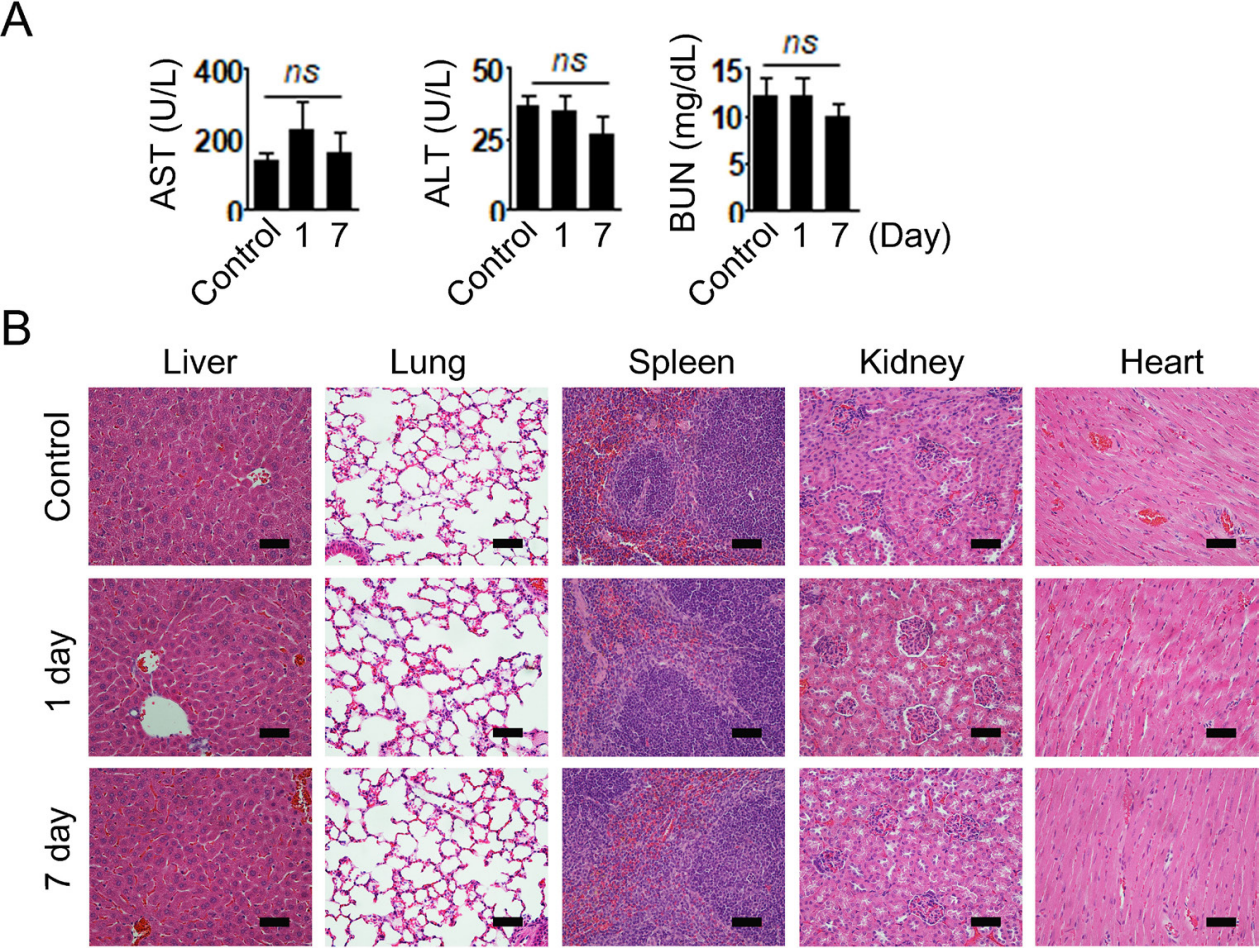
Biochemical toxicity of poly(lactic-co-glycolic acid) (PLGA) (programmed death ligand 1 (PD-L1) small interfering RNA (siRNA)+programmed cell death 1 (PD-1) siRNA)-nanoparticles (NPs). (A) Levels of aminotransferase (AST), alanine aminotransferase (ALT), and blood urea nitrogen (BUN) were evaluated using diagnostic kits in the serum of mice injected with PLGA (PD-L1 siRNA+PD-1 siRNA)-NPs. (B) H&E staining of the major organs (scale bar: 50 μm). Error bars represent the SEM (n=3). ns, not significant.

To evaluate the biochemical toxicity of PLGA (PD-L1 siRNA+PD-1 siRNA)-NPs on liver and renal function, we analyzed the levels of relevant biomarkers for AST, ALT, and BUN in the serum of mice treated with PLGA (PD-L1 siRNA+PD-1 siRNA)-NPs. There were no significant differences in the AST, ALT, and BUN levels between PLGA (PD-L1 siRNA+PD-1 siRNA)-NP-treated group and the control group (figure 7A). Moreover, H&E staining of vital organs was performed to determine the toxicity induced on PLGA (PD-L1 siRNA+PD-1 siRNA)-NP treatment. The histological structures of the organs treated with PLGA (PD-L1 siRNA+PD-1 siRNA)-NPs were similar to those of the control group, indicating no differences in pathological observations (figure 7B).

## Discussion

This study demonstrates the potential of PLGA (PD-L1 siRNA+PD-1 siRNA)-NPs as a novel immunotherapeutic approach for silencing immune checkpoints and a replacement for the existing antibody-based cancer immunotherapies. In the present study, we confirmed the presence of secreted PD-L1 from tumor cells, which is an important issue for the limited use of antibody-based approaches in cancer therapy. To overcome this hurdle, we suggested siRNA-encapsulated PLGA (siRNA)-NP systems and silenced PD-L1 expression in tumor cells, which effectively prevented PD-L1 secretion from tumor cells. Additionally, we demonstrated the therapeutic efficacy of NP-based therapeutic vaccines, which showed significant inhibition of tumor growth to enhance the tumor antigen-specific cytotoxic CD8^+^ T cell response. Consequently, our system provides a synergistic therapeutic potential for cancer immunotherapy.

Cancer immunotherapy is one of the most promising approaches for cancer treatment. Among these approaches, immune checkpoint inhibition using antibodies (anti-PD-1, anti-PD-L1, and anti-CTLA4) offers clinical benefits and can be applied to many cancer types.^36 37^ Although the role of immune checkpoint function via immune evasion mechanisms in the tumor microenvironment is well known, the use of antibody-based blockades has been shown to increase the incidence of acquired resistance to antibodies, and such issues have been reported by several groups.^38 39^ Moreover, effectiveness is only observed in a small fraction of patients, and resistance after initial response is commonly observed.^13^ One major problem that leads to decreasing therapeutic efficacy using anti-PD-L1 or anti-PD-1 is that the immune checkpoint molecules can be released from tumor cells in a soluble form, rather than a membrane-bound form.^12^ In the soluble form, these molecules can suspend and circulate in the tumor microenvironment. Meanwhile, antibody blockades such as anti-PD-L1 and anti-PD-1 can bind to these secreted immune checkpoint molecules, leading to decreased antibody-based therapeutic outcomes.^14 40^ Patients with higher expression of soluble PD-L1 and PD-1 exhibited shorter overall survival rates and tumor-free survival than those with lower expression. These results indicate that patients with higher soluble PD-1 and PD-L1 levels have worse prognosis.^41 42^ These are critical points for the development of therapeutics for broad cancer types. Therefore, we focused on an immune checkpoint silencing system based on siRNA as a novel approach to prevent immune suppressive binding of PD-1 or PD-L1 to anti-PD-1 or anti-PD-L1, respectively. The results of the study demonstrated that there is an increase in therapeutic efficacy on fundamentally inhibiting the secretion of immune checkpoint molecules.

siRNA-based approaches are attractive strategies for knocking-down target molecules.^43^ Moreover, these may allow for the development of a broad medicinal application for silencing target genes. However, one of the hurdles limiting the therapeutic application of siRNA is the need for an efficient delivery system because siRNA alone is rapidly cleared or degraded by nucleases in the body. Recently, NP-based carrier systems have been extensively developed to increase the delivery efficiency of siRNAs. These are highly attractive for cancer therapy because of their specific capacities and biocompatibility. In addition, NPs increase the therapeutic dose at disease sites, thereby minimizing unexpected side effects. In this study, we selected PLGA polymer as an NP matrix, which is particularly attractive for clinical and biological applications, given its low toxicity, low immunogenicity, biocompatibility, and biodegradability.^21^

Combination therapies for cancer have been considered to increase synergistic therapeutic outcomes by integrating two or more therapeutic agents.^44^ As a novel strategy for cancer immunotherapy, a combination of immune checkpoint blockades has been applied to treat many cancer types, which shows greater therapeutic benefit as compared with monotherapies, because it prevents an immunosuppressive response. Based on this study, we propose a combination strategy that uses PLGA (PD-L1 siRNA+PD-1 siRNA)-NPs as an immune checkpoint silencing system alongside NP-based vaccines, which demonstrated promising synergistic effects for the treatment of cancer. NP-based vaccines offer a rational approach to improving safety and reducing the toxicity of adjuvants. In addition, the NP system can further enhance antigen-specific CD8^+^ T cell activation and immune response without increasing toxicity, by allowing for synergy with therapeutic payloads, such as antigens or adjuvants. Therefore, in this study, we used a PLGA (tumor antigen+adjuvant)-NP system in combination with PLGA (PD-L1 siRNA+PD-1 siRNA)-NPs to induce antigen-specific CD8^+^ T cell-based immune responses.^22^

In summary, we focused on the development of an siRNA-based immune checkpoint silencing system to enhance the therapeutic unmet need to improve on antibody-based cancer immunotherapy. Our results provide a novel approach to target gene silencing of immune checkpoints in the tumor microenvironment. Consequently, our study revealed a novel understanding of an siRNA-based cancer immunotherapeutic approach as a nanomedicine platform.

## Data Availability

Data are available in a public, open access repository.
