# Supplementary material for: Immune checkpoint silencing using RNAi-incorporated nanoparticles enhances antitumor immunity and therapeutic efficacy compared with antibody-based approaches
Source: J Immunother Cancer. 2022 Feb 28;10(2):e003928. doi: 10.1136/jitc-2021-003928 (PMC8886443; doi:10.1136/jitc-2021-003928)
Supplement: Supplementary data [file jitc-2021-003928supp001.pdf]

Supplementary Data

# Immune checkpoint silencing using RNAi-incorporated nanoparticles enhances antitumor immunity and therapeutic efficacy compared to antibody-based approaches

Ji Eun Won[1], Yeongseon Byeon[1], Tae In Wi[1], Chan Mi Lee[1], Ju Hyeong Lee[1], Tae Heung Kang[1], Jeong-Won Lee[2], YoungJoo Lee[3], Yeong-Min Park[1,4],and Hee Dong Han[1,4]

## Supplemental Figures

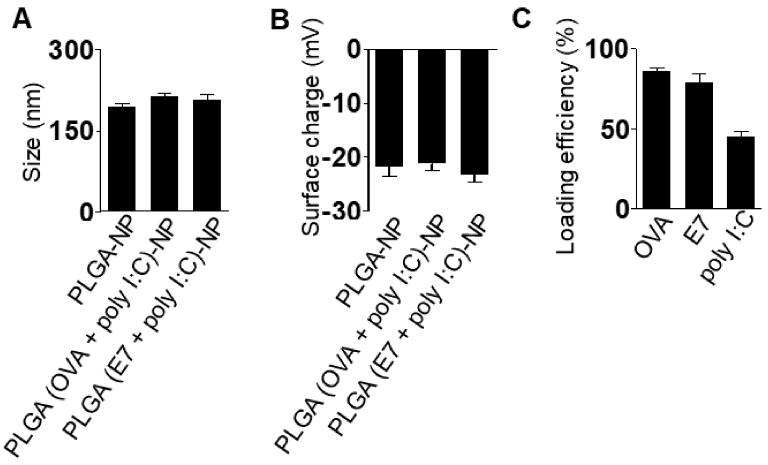


**Supplemental figure S1.** Physical properties of PLGA (antigen + adjuvant)-NPs. **A**, Size and **B**, surface charge of PLGA (OVA + poly I:C)-NPs and PLGA (E7 + poly I:C)-NPs. **C**, Loading efficiency of OVA, E7 peptide, and poly I:C into PLGA (antigen + adjuvant)-NPs. Error bars represent the standard of the mean.

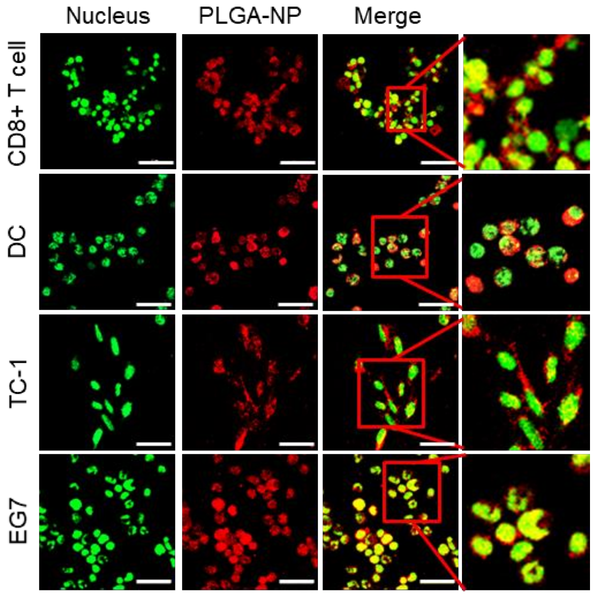


**Supplemental figure S2.** Intracellular delivery efficiency of PLGA (Cy5)-NPs in CD8+ T cells, DCs, TC-1, and EG7 cells. Confocal microscopic images for PLGA (Cy5)-NPs. Red color indicates PLGA-NPs, while green indicates the nuclei of the cells (scale bar: 50 μm).

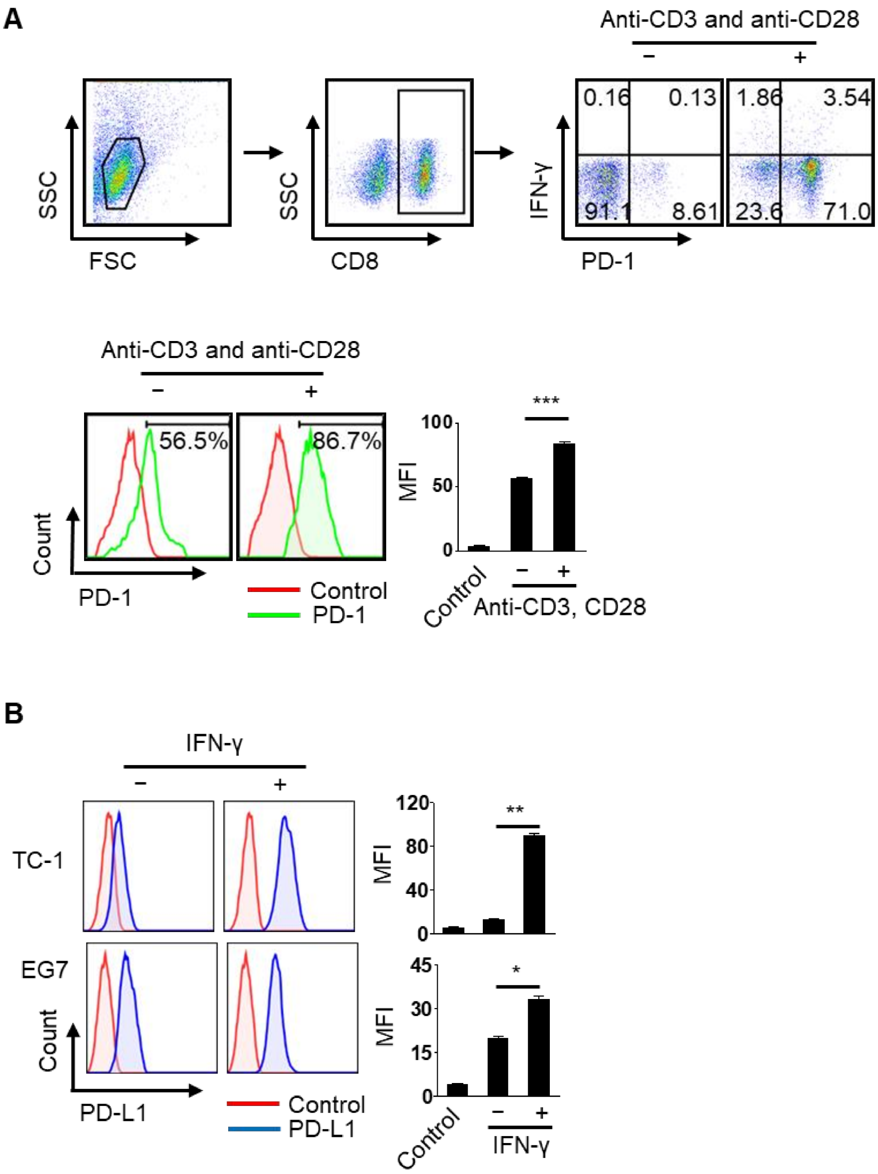


**Supplemental figure S3.** Expression of PD-1 on CD8 + T cells and that of PD-L1 in tumor cells. **A**, CD8+ T cell-gating in splenocytes. PD-1 expression upon anti-CD3 and anti-CD28 stimulation of CD8+ T cells. **B,** PD-L1 expression induced upon IFN-γ stimulation of tumor cells. Error bars represent the standard of the mean. *$p < 0.05$, **$p < 0.01$, and ***$p < 0.001$.

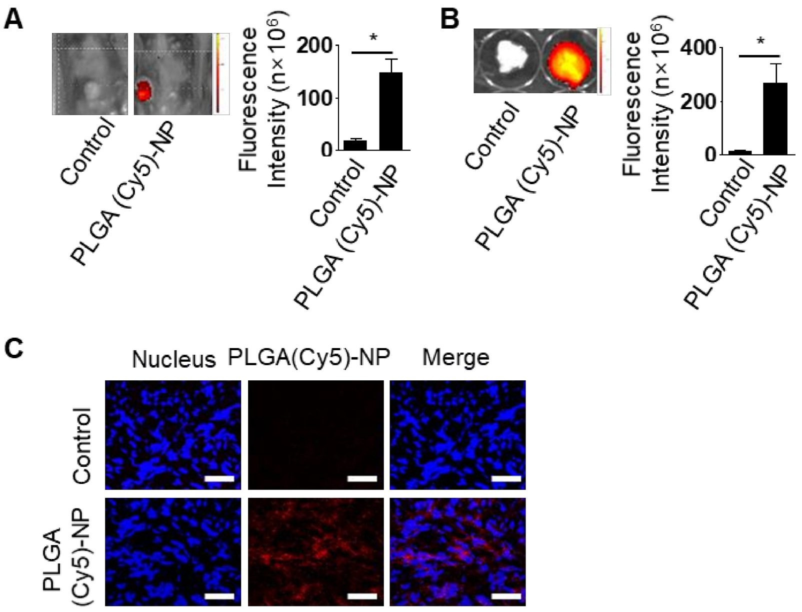


**Supplemental figure S4.** In vivo localization of PLGA (Cy5)-NPs after i.v. injection into TC-1 tumor-bearing mice. Fluorescence images of **A**, mice and **B**, tumor tissues. **C**, Localization of PLGA (Cy5)-NPs (red) in tumor tissues were detected using confocal microscopy. Error bars represent the standard of the mean (scale bar: 50 μm). *$p < 0.05$.

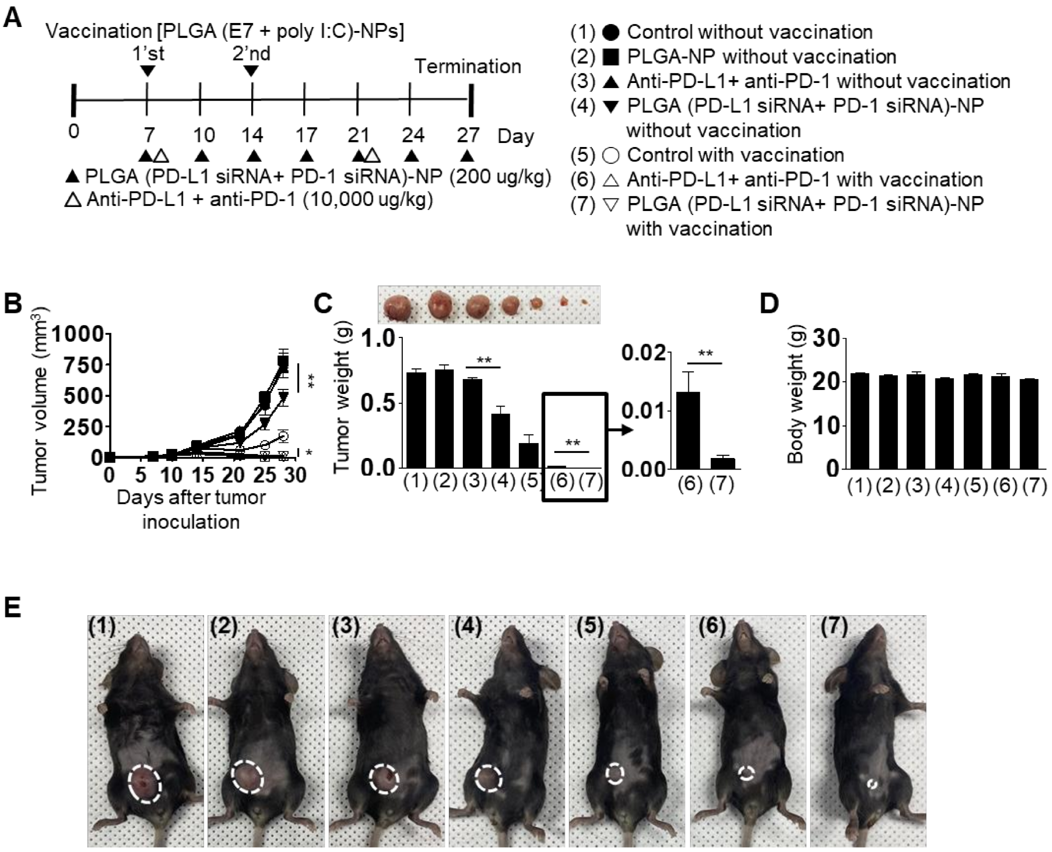


**Supplemental figure S5.** Therapeutic efficacy of antibodies (anti-PD-L1 + anti-PD-1) or PLGA (PD-L1 siRNA + PD-1 siRNA)-NPs against TC-1 tumor-bearing mice. Treatment began one week after s.c. injection of TC-1 cells into the mice. Anti-PD-L1 + anti-PD-1 was injected once every 2 weeks by means of i.v. injection (10,000 µg /kg). PLGA (PD-L1 siRNA + PD-1 siRNA)-NPs were injected twice per week by means of i.v. injection (200 µg/kg). **A**, Experimental schedule for tumor therapy. **B**, Tumor volume, **C**, tumor weight, and **D**, body weight. **E,** Images of treated mice. Error bars represent the standard of the mean. *$p < 0.05$ and **$p < 0.01$.
